# Study on the Community Characteristics of the Endogenous Microbiome in Earthworm Cocoons in Composting Systems with Different Base Materials

**DOI:** 10.3390/microorganisms14071449

**Published:** 2026-06-30

**Authors:** Jinjun Wang, Xinru Gao, Tianyi Jia, Duoduo Chen, Haitao Zhao, Yang Zhang, Jian Hu

**Affiliations:** College of Environmental Science and Engineering, Yangzhou University, Yangzhou 225127, China; jinjunwang@yzu.edu.cn (J.W.); gxr0207@163.com (X.G.); jiatianyi09@gmail.com (T.J.); chenduo0404@163.com (D.C.); htzhao@yzu.edu.cn (H.Z.); 007499@yzu.edu.cn (Y.Z.)

**Keywords:** earthworm cocoons, bacterial communities, composting systems, symbiotic bacteria

## Abstract

This study investigates earthworm cocoons as key vectors for the vertical transmission of symbiotic bacteria, a process that profoundly shapes the gut microbiota of offspring and influences their environmental adaptability. However, systematic knowledge of the internal microbiome communities within earthworm cocoons remains limited. Here, we characterized the composition and functional potential of bacterial communities within cocoons of earthworms collected from three composting systems (fermented coffee grounds, cow manure, and residual sludge) using high-throughput sequencing, together with diversity analyses, dominant taxa identification, and FAPROTAX-based functional prediction. Our results indicated that the composting system significantly affects bacterial diversity and community structure. The fermented coffee grounds system supported the highest species richness, whereas the cow manure system exhibited the greatest diversity and evenness. At the phylum level, Pseudomonadota, Actinomycetota, and Bacteroidota predominated across all systems, with Pseudomonadota being particularly abundant (62.01–81.41%). At the genus level, *Verminephrobacter* and *Agromyces* were consistently dominant, with *Verminephrobacter* showing particularly high relative abundance, ranging from 22.42% to 51.51%. Although the composition and abundance of dominant phyla and genera varied among systems, the shared OTUs accounted for a substantial proportion of the relative abundance in each sample (54.65–91.84%). Functional predictions revealed chemoorganoheterotrophy as the predominant metabolic function, with relative abundances ranging from 23.87% to 45.42%. Collectively, these findings provide insights into how composting environments shape the bacterial communities within earthworm cocoons, offering a theoretical foundation for understanding the ecological functions of earthworms and their potential applications in ecological restoration and sustainable agriculture.

## 1. Introduction

As integral components of soil ecosystems, earthworms facilitate nutrient cycling, improve soil structure, and play a vital role in the biotransformation of organic waste resources. Consequently, they are often regarded as “ecosystem engineers” [1,2]. Earthworm cocoons serve not only as reproductive units but also as crucial habitats for their symbiotic bacteria. The internal microbiome within these cocoons significantly influences offspring growth, reproduction, and environmental adaptability. Therefore, characterizing the structure and function of bacterial communities in earthworm cocoons is essential for elucidating the mechanisms underlying earthworm–bacteria symbiosis in soil and composting systems, as well as for understanding their interactions with environmental factors.

As vermicomposting and earthworm farming receive increasing attention, the role of earthworms throughout agricultural production cycles has gained greater emphasis. Research on the earthworm microbiome has largely centered on the gut and casts, whereas the microbial communities harbored within earthworm cocoons remain comparatively understudied. Davidson and colleagues [3,4,5] employed fluorescence probe techniques to demonstrate that typical symbiotic bacteria of *Eisenia fetida* are abundantly deposited and retained within earthworm cocoons and subsequently colonize the intestinal lumen of newly hatched juveniles. These bacteria play important roles in nutrient decomposition, metabolic waste processing, and immune regulation [6]. Earlier studies based on traditional culturing and microscopic observation have also revealed diverse and abundant bacterial communities within earthworm cocoons [7,8]. Aira et al. [9] reported that the bacterial communities within cocoons of *Eisenia andre* and *Eisenia fetida* were predominantly composed of Pseudomonadota, Bacteroidota, and Actinomycetota, with *Verminephrobacte* being detected in both species. In addition, Lund et al. [10,11] found that typical earthworm symbionts, including *Verminephrobacter* and *Flexibacter*, are commonly present in earthworm cocoons and play a promoting role in host reproduction. However, aside from a few well-characterized symbiotic bacteria, the mechanisms by which the majority of these bacteria are enriched within the cocoons, as well as their migration and functional roles during development from egg to juvenile earthworm, remain poorly understood.

In recent years, high-throughput sequencing technologies have greatly facilitated the efficient study of environmental bacterial communities and their functions. Nevertheless, few studies have applied these techniques to investigate the internal microbiome communities and functional characteristics of earthworm cocoons, or to explore their relationships with environmental factors. In this study, we used 16S rRNA high-throughput sequencing to analyze the composition of bacterial communities within earthworm cocoons from different composting systems. Our objectives were to characterize the functional attributes of these internal microbiome communities, compare their shared features across distinct composting environments, and examine their associations with environmental factors. This work provides a foundation for further clarifying the migration and transmission roles of these bacteria throughout the earthworm life cycle.

## 2. Materials and Methods

### 2.1. Source of Earthworm Cocoons 

Earthworm cocoons were collected from vermicomposting facilities utilizing excess sludge and cow manure as substrates—specifically, Xinhuan Ecological Agriculture Co., Ltd. (Xinhuan, China), which employs open-air cultivation, and Kangsheng Ecological Agriculture Development Co., Ltd. (Changzhou, China), which uses greenhouse cultivation—as well as from a laboratory-scale vermicomposting system established in our laboratory, where fermented coffee grounds (processed with Trichoderma harzianum) were used as the substrate. All earthworms belonged to the species *Eisenia fetida* (commonly known as the Japanese tiger worm, commercial variety “Daping No. 2”).

Preparation of fermented coffee residue: The fermented coffee residue substrate for *Trichoderma harzianum* fermentation was prepared, given the nutrient-rich nature of spent coffee grounds. Add 6% wheat bran by weight, 2% molasses solution by volume, and 3% *Trichoderma harzianum* culture solution to the coffee residue. Adjust the moisture content to 65% using purified water. After thorough mixing, portion the coffee residue into 6-litre lidded plastic containers (25 cm × 17.5 cm × 13.5 cm) at 2 kg per container. Immediately subject these to high-pressure steam sterilisation (121 °C, 60 min). After cooling, open containers within a laminar flow hood. Add 3% inoculum of *Trichoderma harzianum* solution, followed by three layers of sterile gauze. Fermentation then proceeds naturally at room temperature.

### 2.2. Experimental Design and Treatment Coding

Treatment groups were systematically designated based on the origin of the earthworm cocoons: coffee ground compost (g0), sludge compost (s0), and cow manure compost (m0).

### 2.3. Sample Collection and Reagent Preparation

PBS buffer (pH 7.20–7.40) was prepared by dissolving 7.90 g NaCl, 0.20 g KCl, and either 0.24 g KH_2_PO_4_ or a combination of 1.44 g Na_2_HPO_4_ and 1.80 g K_2_HPO_4_ in approximately 800 mL of deionized water, adjusting the pH to 7.40 with HCl, and bringing the volume to 1 L with deionized water. PBS-S buffer (phosphate-buffered saline containing 0.02% *v*/*v* Silwet-77) was obtained by filter-sterilizing the Silwet-77 stock solution and adding it to the prepared PBS buffer.

For washing, earthworm cocoons were placed in 2 mL centrifuge tubes and immersed in PBS-S solution. The tubes were shaken on an orbital shaker at 180 rpm for 20 min, after which the PBS-S was discarded. Fresh PBS-S was added to submerge the samples again, followed by another 20 min of shaking at 180 rpm. After removing the PBS-S from this second wash, new PBS-S was added to cover the samples. The tubes were then placed on a tube holder and submerged in an ultrasonic cleaner, with the water level exceeding the height of the sample material in the tubes. Ultrasonic cleaning was performed at 50–60 Hz with a 30 s pulse. This step was repeated twice with fresh PBS-S, resulting in a total of three ultrasonic washes. Finally, the cocoons were collected and stored at −80 °C for subsequent bacterial DNA extraction from within the cocoons.

### 2.4. DNA Extraction and Illumina MiSeq Sequencing Analysis

Total DNA was extracted from the samples using the stDNA^®^ SPIN Kit for soil (MP Biomedicals, Santa Ana, CA, USA). DNA concentration was measured with an Epoch Take3™ microplate reader, and integrity was verified by 2% agarose gel electrophoresis. Qualified DNA samples were stored at −20 °C until further use.

The 16S rRNA gene was amplified from different samples using the universal primer pair 799f (5′-AACMGGATTAGATACCCKG-3′) and 1193r (5′-ACGTCATCCCCACCTTCC-3′) [12]. Each primer contained a unique adapter sequence. The PCR reaction mixture (20 μL total volume) consisted of a 4 μL 5× FastPfu Buffer, 2 μL 2.50 mM dNTPs, 0.8 μL each of forward and reverse primers (5 μM), 0.4 μL FastPfu Polymerase, and 10 ng template DNA, with the remaining volume made up of ddH_2_O. The thermal cycling conditions were as follows: initial denaturation at 98 °C for 1 min; 30 cycles of 98 °C for 15 s, 55 °C for 30 s, and 72 °C for 30 s; and a final extension at 72 °C for 10 min.

PCR products were purified and subsequently sent to Shanghai Personal Biotechnology Co., Ltd. (Shanghai, China) for high-throughput sequencing. Paired-end sequencing (2 × 250 bp) was performed on an Illumina NovaSeq platform.

### 2.5. Bioinformatic Analysis

Raw sequencing data were processed using QIIME2 (version 2024.10) following the pipeline below: 1. Adapter and primer sequences were trimmed from raw reads with the cutadapt plugin. 2. Quality filtering, denoising, paired-end merging, and chimera removal were performed using DADA2, generating a feature table and representative sequences of amplicon sequence variants (ASVs, equivalent to operational taxonomic units, OTUs). 3. Representative sequences of each OTU were taxonomically annotated by comparison against the RDP database with a confidence threshold of 0.80. 4. To compare bacterial community diversity across samples, all samples were rarefied to the minimum read count among the sequenced samples10. 

### 2.6. Statistical Analysis

Statistical analysis was conducted using SPSS 19.0 with a significance level of *p* < 0.05 in general linear models. Bar plots were generated with Origin 2025. Bar charts and box plots were generated using Origin 2024. For community analysis, a phylogenetic tree was employed to reflect the evolutionary relationships among microbial species within the community, a clustered heatmap was plotted using the “pheatmap” package, and a Venn diagram at the OTU level was generated using the “CNS” platform. Functional prediction of bacterial communities in earthworm samples was carried out with FAPROTAX via the online platform LingEn Micro-Class (http://www.cloud.biomicroclass.com/CloudPlatform/SoftPage/FAP, accessed on 3 June 2026), and results were visualized using the “ggplot2” package.

## 3. Results

### 3.1. Alpha Diversity of Internal Microbiome Communities in Earthworm Cocoons from Different Composting Systems

**Figure 1:**
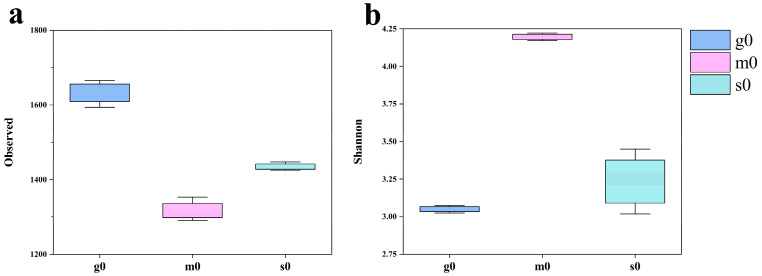
α-diversity indices of internal microbiome communities in earthworm cocoons from different composting systems. (Note: g0: earthworm cocoons from fermented coffee grounds compost, m0: earthworm cocoons from cow dung compost, s0: earthworm cocoons from sludge compost; the same below).

The alpha diversity of the internal microbiome in earthworm cocoons derived from fermented coffee grounds (g0), cattle manure (m0), and sewage sludge (s0) is shown in Figure 1. The observed species index followed the order g0 > s0 > m0 (Figure 1a), indicating that the total number of bacterial species was highest in cocoons from the fermented coffee grounds system. In contrast, the Shannon index exhibited the pattern m0 > s0 > g0 (Figure 1b), suggesting that the bacterial community in the cattle manure system not only had higher diversity but also a more even species distribution.

Notably, while the g0 treatment showed a significantly higher observed species index than the m0 treatment, the opposite trend was observed for the Shannon index. This seemingly contradictory result actually reflects distinct ecological characteristics of the communities: although the fermented coffee grounds system harbored a greater number of species (higher richness), the distribution of these species was likely uneven, potentially dominated by a few predominant taxa. In comparison, the cattle manure system, despite having fewer total species, exhibited a more balanced distribution among taxa, thereby enhancing overall community diversity.

### 3.2. Beta Diversity of Internal Microbiome Communities in Earthworm Cocoons Across Composting Systems

**Figure 2:**
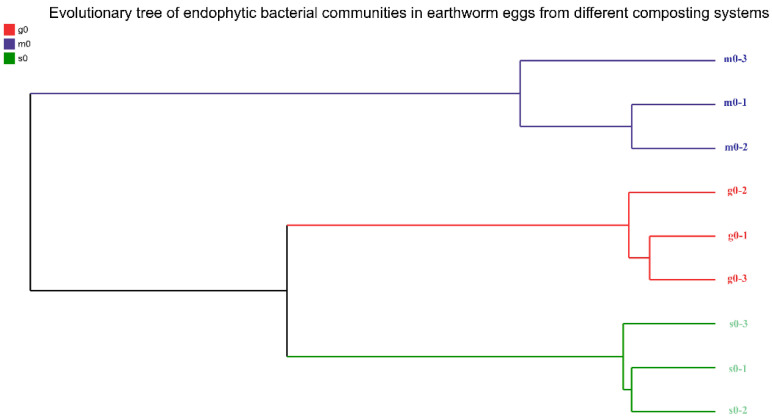
Phylogenetic relationships among microbial taxa within internal microbiome communities in earthworm cocoons across different composting systems.

The results of internal microbiome communities in earthworm cocoons derived from different composting systems are shown in Figure 2. As illustrated, the bacterial communities of g0, m0, and s0 are distinctly separated in the ordination space, indicating clear structural differences among the internal microbiome communities of cocoons produced under fermented coffee grounds, cattle manure, and sludge vermicomposting systems.

### 3.3. Analysis of Dominant Bacterial Taxa in Earthworm Cocoons from Different Composting Systems

**Figure 3:**
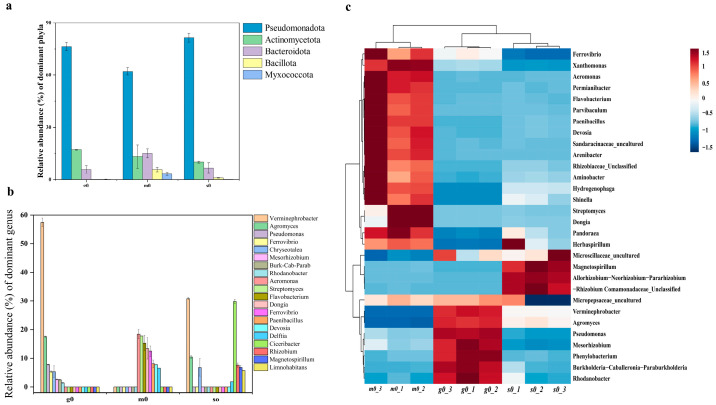
(**a**): Relative abundance of major bacterial phyla of the internal microbiome in earthworm cocoons in different composting systems. (**b**): Relative abundance of major bacterial genera in earthworm cocoons from different composting systems. (**c**): Cluster analysis of dominant genera in earthworm cocoons from different composting systems.

The relative abundances of the top 5 bacterial phyla and the top 20 bacterial genera in the internal microbiome communities of earthworm cocoons from different composting systems were statistically analyzed, with results presented in Figure 3a.

As shown in Figure 3a, Pseudomonadota, Actinomycetota, Bacteroidota, and Bacillota were the dominant phyla across the cocoons’ bacterial communities, with relative abundances ranging from 62.18% to 81.49%, 10.07% to 17.06%, 5.79% to 15.11%, and 0.18% to 5.80%, respectively. In cocoons from the fermented coffee grounds system, Pseudomonadota exhibited a higher abundance, whereas Bacteroidota and Actinomycetota were relatively lower. Compared with those from fermented coffee grounds, cocoons from the cattle manure system showed reduced abundances of Pseudomonadota and Actinomycetota, but increased abundances of Bacteroidota and Bacillota. Notably, the highest relative abundance of Myxococcota was observed in the cattle manure system. In the sludge system, Pseudomonadota reached its highest abundance, while Actinomycetota, Bacteroidota, and Bacillota decreased compared with the cattle manure system.

These results indicate that, at the phylum level, Pseudomonadota, Actinomycetota, and Bacteroidota were consistently present as dominant phyla in earthworm cocoons across different composting systems. Pseudomonadota maintained a notably high relative abundance in all systems. Nevertheless, both the composition and abundance levels of the dominant bacterial phyla varied to different degrees among the composting systems.

As illustrated in Figure 3b, the dominant bacterial genera in the cocoon communities mainly included *Verminephrobacter*, *Ciceribacter, Agromyces*, and *Aeromonas*, with relative abundances ranging from 2.42% to 57.43%, 0.00% to 29.08%, 0.00% to 17.54%, and 0.00% to 18.43%, respectively. The bacterial community composition differed among the three composting systems (g0, m0, and s0), suggesting that the composting system may influence the structure of the bacterial community within earthworm cocoons.

A cluster analysis was further performed on the dominant bacterial genera in earthworm cocoons across the composting systems (Figure 3c). The results showed that the systems were clustered into two major groups: one consisting of m0 and the other comprising g0 and s0. This indicates that, at the genus level, the internal microbiome community in cocoons from the cattle manure system differs considerably from those in the fermented coffee grounds and sludge systems, while the latter two systems exhibit a high degree of similarity.

Together, these findings demonstrate that at the genus level, *Verminephrbacter*, *Agromyces*, and other dominant genera are present in earthworm cocoons across all composting systems. Although *Verminephrobacter* consistently maintained a high relative abundance regardless of the system, both the composition and abundance levels of the dominant bacterial genera still varied to different extents among the different composting systems.

### 3.4. Analysis of Shared Characteristics of Internal Microbiome Communities in Earthworm Cocoons from Different Composting Systems

**Figure 4:**
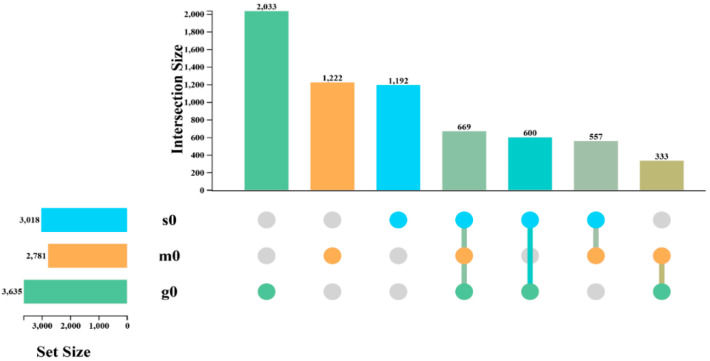
Venn diagram of OTUs distribution of internal microbiome in earthworm cocoons in different composting systems.

As shown in Figure 4, the total numbers of OTUs in g0, m0, and s0 were 3635, 2781, and 3018, respectively. A total of 669 OTUs were shared among all three groups, accounting for 91.84%, 54.67%, and 54.65% of the total OTUs in each corresponding sample. In contrast, the numbers of unique OTUs in g0, m0, and s0 were 2033, 1222, and 1192, respectively, with their cumulative relative abundances within each sample being 3.96%, 2.73%, and 2.00%.

These results indicate that although the number of unique OTUs in the internal microbiome communities of earthworm cocoons clearly exceeds the number of shared OTUs across the different composting systems, the shared OTUs contribute substantially to the relative abundance within each sample. This suggests that while the composting substrate may influence the formation of the internal microbiome community in earthworm cocoons, the high degree of OTU sharing reflects considerable community stability across systems.

### 3.5. Functional Characteristics of Internal Microbiome Communities in Earthworm Cocoons from Different Composting Systems

**Figure 5:**
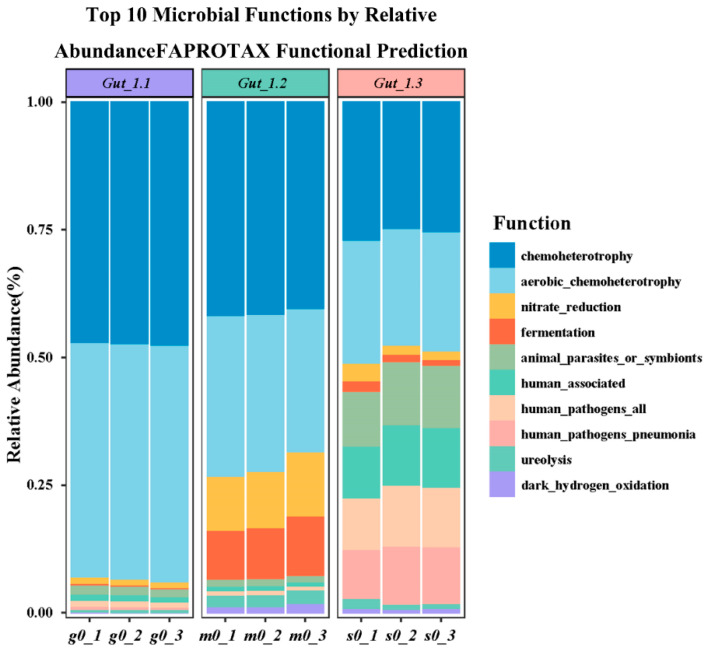
Relative abundance of main functional potentials of endophytes in earthworm cocoons in different composting systems.

FAPROTAX [13] is a prokaryotic environmental function database widely used for predicting functional potentials of bacterial communities in environmental samples, including roles in biogeochemical cycling, metabolism, and fermentation processes. Based on the 16S rRNA gene sequencing data obtained from earthworm cocoons across the three composting systems in this study, FAPROTAX analysis predicted a total of 74 functional groups. The top 10 most abundant functional groups are shown in Figure 5.

As shown in Figure 5, the dominant predicted functions of the bacterial communities in earthworm cocoons across different composting systems include chemoheterotrophy, aerobic chemoheterotrophy, nitrate reduction, and fermentation, with relative abundances ranging from 23.87% to 45.42%, 21.82% to 44.72%, 0.72% to 10.01%, and 0.62% to 9.76%, respectively. Among these, chemoheterotrophy—particularly aerobic chemoheterotrophy—exhibited consistently high abundance in the internal microbiome of earthworm cocoons from all composting systems.

**Figure 6:**
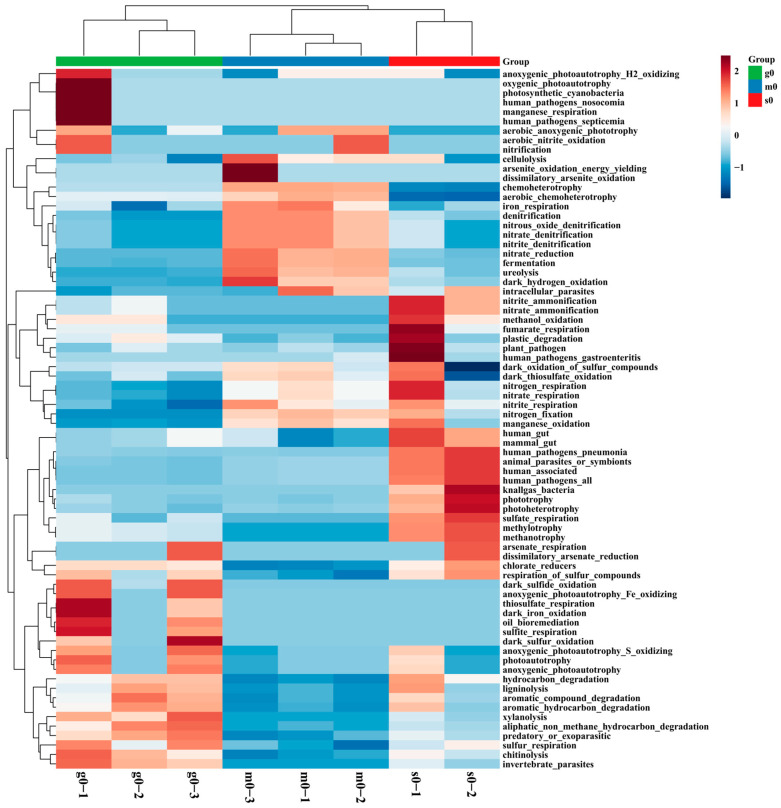
Heatmap of functional abundance in the internal microbiome communities of earthworm cocoons from different composting systems.

Based on heatmap analysis of the similarity in the relative abundance of predicted functions among the three types of earthworm cocoons’ internal microbiome, the results are shown in Figure 6. The FAPROTAX ecological functions of the bacterial communities from the three composting systems are clustered into two groups: one comprising g0 and the other containing m0 and s0. This indicates that the functional profile of the internal microbiome community in earthworm cocoons from the fermented coffee grounds system differs considerably from those of the cattle manure and sludge systems, whereas the cattle manure and sludge systems exhibit relatively high functional similarity.

Furthermore, the relative abundances of the predicted functions in the bacterial communities of earthworm cocoons from different composting systems were categorized into three levels for comparative analysis: high (>1.0%), medium (0.1–1.0%), and low (<0.1%). The results are presented in Table 1, Table 2 and Table 3.

As shown in Table 1, significant differences were generally observed in high-abundance functions across the three composting systems, indicating that the composting substrate influences the predominant functional potentials of the bacterial communities within earthworm cocoons. Table 2 shows that, overall, the medium-abundance functional profiles did not differ markedly among the composting systems, suggesting that these functions are less affected by the substrate and maintain relatively stable functional potential across different composting materials. According to Table 3, low-abundance functional traits generally showed minimal variation across the three composting systems. 

These results demonstrate that high-abundance functions (e.g., chemoheterotrophy, aerobic chemoheterotrophy, and nitrate reduction) differed significantly among the three composting systems, whereas medium- and low-abundance functions (e.g., ureolysis, dark hydrogen oxidation, and manganese oxidation) exhibited only minor differences between systems.

## 4. Discussion

### 4.1. Variation in Internal Microbiome Community Diversity Among Different Composting Systems

The diversity and composition of internal microbiome communities in earthworm cocoons varied across the composting systems. In terms of alpha diversity, the fermented coffee grounds system exhibited the highest observed species index, indicating the greatest species richness. Fermented coffee grounds contain a wide range of complex organic compounds, such as polysaccharides, proteins, and phenolic substances. The decomposition of polysaccharides, in particular, can provide carbon sources for numerous bacteria, allowing taxa with preferences for different sugars to occupy suitable ecological niches [14,15], thereby promoting higher species richness. 

In contrast, the cattle manure system showed the highest Shannon index, reflecting the highest bacterial diversity and a relatively even community structure. This is likely attributable to the fact that cattle manure contains not only major nutrients such as nitrogen, phosphorus, and potassium, but also diverse trace elements including calcium, magnesium, and zinc [16,17]. Such nutritional diversity supplies ample mineral nutrients for various bacterial groups. Moreover, cattle manure exhibits relatively stable physicochemical properties [18] and effectively improves microbial habitat conditions. As an excellent inorganic amendment, it demonstrates notable advantages in carbon sequestration and nutrient recovery, contributing to the regulation and maintenance of a more stable micro-ecological environment. This suitable habitat further stimulates the activity of functional microorganisms associated with metabolic processes, ultimately enhancing the efficiency of organic matter biodegradation [19].

### 4.2. Characteristics of the Dominant Bacterial Community in Earthworm Cocoons and Their Ecological Adaptability in Different Composting Systems

From the phylum level, Pseudomonadota, Actinomycetota, Bacteroidota, Bacillota, etc., are the dominant bacterial phyla in the three composting systems, but their relative abundances show significant differences among the different composting systems.

Pseudomonadota, as the dominant phylum shared among the three composting systems, is known to enhance earthworms’ decomposition and utilization of organic matter [20,21], produce various antimicrobial substances that help resist pathogen invasion, and improve disease resistance [21]. Its highest relative abundance in the fermented coffee grounds substrate may be attributed to the presence of complex organic compounds such as polycyclic aromatic hydrocarbons, which provide a survival advantage for pseudomonads [22]. Bacteroidota, commonly found in the earthworm gut, can influence soil microbial community structure through direct feeding and excretion, thereby increasing their relative abundance in soil [23]. Additionally, they exhibit high activity under anaerobic or low-oxygen conditions and participate in fermentation processes within the earthworm gut, supplying essential energy and nutrients for earthworm life activities [24]. Bacteroidota and Bacillota showed higher abundances in the cattle manure system, likely due to the rich cellulose content in cattle manure. Bacillota, which frequently decomposes lignocellulosic components in compost, is widely used as a plant growth promoter and also serves as an important agent in bioremediation. It is commonly found in contaminated environments, not only tolerating pollutants and participating in remediation processes but also efficiently degrading petroleum hydrocarbons, aromatic compounds, and textile dyes [25]. In the cattle manure system, Myxococcota secretes various carbohydrate-active enzymes that efficiently degrade plant residues and lignocellulose, promoting the cycling of carbon, nitrogen, and other elements in soil and positively contributing to ecosystem productivity [26,27].

At the genus level, *Verminephrobacter*, *Ciceribacter*, *Agromyces*, and *Aeromonas* were the dominant genera across different composting systems. Among them, *Verminephrobacter* is a typical symbiotic bacterium widely present in earthworm cocoons and plays important roles in nutrient decomposition, metabolic waste processing, and immune regulation [6,28]. Early studies using fluorescence in situ hybridization have revealed the enrichment of *Verminephrobacter* within earthworm cocoons and its migration and colonization into the gut lumen of juvenile earthworms [3]. This bacterium helps juvenile earthworms rapidly establish a stable gut microbiota, thereby enhancing their survival capacity and adaptability [29,30,31]. Ciceribacter showed the highest relative abundance in the sludge composting system, possibly because sludge contains elevated levels of heavy metals and complex organic compounds [32,33], which select for unique microbial traits. Ciceribacter may possess heavy-metal tolerance and the ability to degrade complex organic substances [34], giving it a competitive advantage in the sludge system. *Agromyces* was most abundant in the fermented coffee grounds system, likely due to the presence of polycyclic aromatic hydrocarbons and other complex organics that provide a niche for this genus [22]. *Aeromonas* exhibited the highest relative abundance in the cattle manure system, which may be explained by its strong cellulose-degrading capacity [35] and the abundant cellulose content in cattle manure that serves as a rich nutrient source.

Further cluster analysis of dominant bacterial genera in earthworm cocoons from different composting systems revealed that the samples were grouped into two major clusters: one comprising m0 and the other comprising g0 and s0. This indicates that the internal microbiome community in earthworm cocoons from the cow manure composting system (m0) differs substantially from those in the fermented coffee grounds (g0) and sludge (s0) systems at the genus level, whereas the latter two systems exhibit a high degree of similarity.

Overall, differences in the physicochemical properties of composting substrates lead to adaptive shifts in the internal microbiome communities of earthworm cocoons, ensuring that hatched offspring possess enhanced environmental adaptability.

### 4.3. High Sharing of Internal Microbiome Communities in Earthworm Cocoons and the Ecological Significance of Their Functional Prediction

The Venn diagram analysis revealed that the total number of OTUs in the internal microbiome communities of earthworm cocoons differed across composting systems, with the number of shared OTUs being significantly lower than that of unique OTUs. However, the shared OTUs consistently accounted for a high proportion of the relative abundance in each sample, indicating that while the composting substrate influences the composition of the internal microbiome community, the community structure remains highly stable. This high degree of sharing suggests that the earthworm–bacterial symbiotic association possesses a certain level of conservation. It may represent the core gut microbiota of earthworms, potentially involved in growth, metabolism, reproduction, and environmental adaptation, thereby serving as an essential foundation for maintaining normal physiological functions and species continuity [36,37]. 

Based on FAPROTAX functional prediction analysis, the internal microbiome communities within earthworm cocoons from different composting systems exhibited distinct environmental adaptive characteristics in metabolic functions. Their core metabolic processes were dominated by chemoheterotrophy, aerobic chemoheterotrophy, nitrate reduction, and fermentation, reflecting long-term evolutionary adaptation to organic-rich environments. Notably, although these core functions were universally present, their relative abundances varied significantly among the systems, suggesting that different composting environments exerted specific selective pressures on the functional profiles of the communities.

Specifically, the bacterial community in the fermented coffee grounds system showed the highest abundance of aerobic chemoheterotrophy, indicating a potentially stronger capacity for aerobic organic matter decomposition. In contrast, fermentation was most prominent in the cattle manure system, suggesting that its community may be better adapted to low-oxygen or anaerobic metabolic environments [38]. This functional divergence is closely linked to the physicochemical properties of each composting system—the loose structure of coffee grounds likely promotes aerobic conditions, whereas the dense texture of cattle manure may create more micro-anaerobic niches.

From the perspective of functional abundance hierarchy, high-abundance functions were most sensitive to environmental factors, while medium- and low-abundance functions remained relatively conserved across systems, possibly representing a foundational functional reservoir of the cocoons’ bacterial community. Of particular interest is the widespread presence of nitrate reduction and fermentation functions, which not only reflects the potential role of these communities in nitrogen cycling but also suggests their involvement in the initial decomposition of organic matter under the anaerobic conditions of the earthworm gut.

Collectively, these findings lead to the conclusion that the properties of the composting substrate profoundly shape the metabolic functional landscape of the internal microbiome community in earthworm cocoons by selecting specific functional microbial groups. Such functional differentiation ensures that core metabolic demands are met while simultaneously endowing earthworms in different systems with the metabolic flexibility to respond to specific environmental challenges.

## 5. Conclusions

This study demonstrates that composting substrate type significantly shapes the diversity, composition, and functional potential of internal microbiome communities in earthworm cocoons. Fermented coffee grounds promoted the highest species richness, while cattle manure supported greater diversity and evenness. Pseudomonadota, Actinomycetota, and Bacteroidota were the dominant phyla, with *Verminephrobacter* and *Agromyces* as core genera—*Verminephrobacter* maintained high relative abundance (22.42–51.51%) across all systems. Shared OTUs accounted for 54.65–91.84% of community abundance, indicating high stability despite environmental variation. Functional predictions showed that dominant metabolic functions varied among systems, whereas low-abundance functions remained conserved. Collectively, these findings reveal that composting substrates and the earthworm host jointly regulate cocoons microbiome assembly, providing a theoretical basis and practical support for applying earthworm-associated microbial resources in ecological restoration and sustainable agriculture.

## Figures and Tables

**Table 1:** High-abundance functions (relative abundance > 1.00%) in the internal microbiome communities of earthworm cocoons from different composting systems.

Function Name	g0 (%)	m0 (%)	s0 (%)
Chemoheterotrophy	45.42 a	40.81 b	23.87 c
Aerobic Chemoheterotrophy	44.72 a	30.58 b	21.82 c
Nitrate Reduction	0.72 a	10.01 b	2.68 c
Fermentation	0.62 a	9.76 b	2.38 c
Animal Parasites or Symbionts	1.20 a	0.96 a	9.32 b
Human-Associated	1.11 a	0.94 a	9.28 b
Human Pathogens All	1.07 a	0.93 a	9.16 b
Human Pathogens Pneumonia	0.86 a	0.33 b	8.71 c
Ureolysis	0.20 a	1.77 b	1.31 c
Dark Hydrogen Oxidation	0.23 a	1.30 b	0.91 c
Nitrogen Fixation	0.02 a	0.50 b	1.06 c

Note: Different lowercase letters following data within the same row and category indicate significant differences between treatments, *p* < 0.05.

**Table 2:** Medium-abundance functions (0.10% ≤ relative abundance ≤ 1.00%) in the internal microbiome communities of earthworm cocoons from different composting systems.

Function Name	g0 (%)	m0 (%)	s0 (%)
Methylotrophy	0.07 a	0.01 b	0.16 c
Methanotrophy	0.07 a	0.01 b	0.16 c
Hydrocarbon Degradation	0.23 a	0.02 b	0.38 a
Phototrophy	0.05 a	0.03 a	0.30 b
Aromatic Compound Degradation	0.17 a	0.03 b	0.24 a
Chlorate Reducers	0.18 a	0.02 b	0.16 a
Invertebrate Parasites	0.07 a	0.00 b	0.02 a
Respiration of Sulfur Compounds	0.05 a	0.01 a	0.04 a
Predatory or Exoparasitic	0.05 a	0.01 b	0.02 a
Denitrification	0.02 a	0.02 a	0.02 a
Nitrate Denitrification	0.02 a	0.02 a	0.02 a
Nitrite Denitrification	0.02 a	0.02 a	0.02 a
Nitrous Oxide Denitrification	0.02 a	0.02 a	0.02 a
Sulfur Respiration	0.03 a	0.01 a	0.01 a
Sulfate Respiration	0.01 a	0.00 a	0.03a
Manganese Oxidation	0.07 a	0.33 a	0.84 a
Intracellular Parasites	0.11 a	0.06 a	0.06 a
Dark Thiosulfate Oxidation	0.10 a	0.06 a	0.14 a
Human Gut	0.04 a	0.02 a	0.10 b
Mammal Gut	0.04 a	0.02 a	0.10 b
Plastic Degradation	0.05 a	0.02 a	0.18 b

Note: Different lowercase letters following data within the same row and category indicate significant differences between treatments, *p* < 0.05.

**Table 3:** Low-abundance functions (relative abundance < 0.10%) in the endophytic bacterial communities of earthworm cocoons from different composting systems.

Function Name	g0 (%)	m0 (%)	s0 (%)
Aliphatic Non Methane Mydrocarbon Degradation	0.09 a	0.01 b	0.07 a
Iron Respiration	0.07 a	0.04 a	0.02 a
Cellulolysis	0.04 a	0.03 a	0.03 a
Chitinolysis	0.08 a	0.01 b	0.04 a
Nitrite Respiration	0.02 a	0.02 a	0.03 a
Photoautotrophy	0.02 a	0.01 a	0.01 a
Anoxygenic Photoautotrophy	0.02 a	0.01 a	0.01 a
Anoxygenic Photoautotrophy S Oxidizing	0.02 a	0.01 a	0.01 a
Nitrite Ammonification	0.00 a	0.00 a	0.02 a
Dark Sulfide Oxidation	0.02 a	0.00 a	0.00 a
Anoxygenic Photoautotrophy H_2_ Oxidizing	0.01 a	0.01 a	0.00 a
Human Pathogens Gastroenteritis	0.00 a	0.00 a	0.02 a
Methanol Oxidation	0.00 a	0.00 a	0.01 a
Oil Bioremediation	0.01 a	0.00 a	0.00 a
Fumarate Respiration	0.00 a	0.00 a	0.01 a
Aerobic Anoxygenic Phototrophy	0.01 a	0.01 a	0.00 a

Note: Different lowercase letters following data within the same row and category indicate significant differences between treatments, *p* < 0.05.

## Data Availability

The original data presented in the study are openly available in the Genome Sequence Archive of the China National Center for Bioinformation (accession number PRJCA052629).
